# Prevalence and Predictors of Domestic-Violence towards Wives by their Psychiatric Hospitalized Husbands

**Published:** 2015-09

**Authors:** Ali Sahraian, Ahmad Ghanizadeh, Seyed Hamzeh Hashemi, Mohammad Reza Mohammadi, Laaya Ahmadzadeh

**Affiliations:** 1 Research Center for Psychiatry and Behavioral Sciences, Shiraz University of Medical Sciences, Hafez Hospital, Shiraz, Iran; 2 Department of Psychiatry, Hafez Hospital, School of Medicine, Shiraz University of Medical Sciences, Shiraz, Iran; 3 Department of Neurosciences, School of Advanced Medical Sciences and Technologies, Shiraz University of Medical Sciences, Shiraz, Iran; 4 Psychiatry and Psychology Centre, Department of Psychiatry, Roozbeh Hospital, Tehran University of Medical Sciences, Tehran, Iran

**Keywords:** *Husband*, *Wife*, *Domestic Violence*, *Patient*, *Psychiat*

## Abstract

**Objective: **Violence imposed on wives by their inpatient psychiatric husbands has not been studied yet. The current study surveyed the rates and predictors of violence committed by inpatient psychiatric husbands towards their wives.

**Methods: **A convenient sample of wives of 209 married male psychiatric inpatients completed a self-reported questionnaire. They were asked about physical, emotional, social and economic abuse.

**Results:** More than 80% of the husbands socially abused their wives; 73.0% of the wives had been regularly beaten by their husbands; the rate for humiliation was 77.2%; and only 14.1% of the wives reported that their sexual relationship with their husbands is with desire.

**Conclusion** There is a dramatic high rate of different types of abuse toward wives by their inpatient psychiatric husbands. They are commonly victimized by their husbands. Moreover, different types of violence always co-occur. Future studies should consider this important issue which is unfortunately an ignored research area.

The victimization of women by their husbands is not uncommon. There are many reports from different parts of the world indicating the high rate of domestic violence. Meanwhile, the consequences of violence towards wives are not limited to women. Partner domestic violence negatively affects children reducing children's growth (1).

Domestic violence is limited to physical abuse. There are many other forms of domestic violence such as emotional, sexual and economic violence. Iran is located in Middle East, Asia; and high rates of abuse against women are reported from this part of the world.

A cross-sectional survey from Nepal including 1,296 married women showed that 46% of them had experienced sexual violence at some times in their lives. Moreover, 31% had experienced sexual violence during the past 12 months. Contrary to husbands’ educational level, the educational levels of wives was not a protective factor (2). A study from China reported that 24.6% of the women experienced psychological aggression during the last year. The rate for physical assaults was 5.5% (3).

Self-reported past-year and lifetime prevalence of physical violence in a community-based study from Pakistan was 56.3% and 57.6%, respectively. The rates for sexual violence were 53.4% and 54.5%, respectively. Moreover, psychological abuse was very common with the rates of 81.8% and 83.6%, respectively (4). The risk factors of physical abuse were as follows: Husband’s low educational level, unskilled worker status, and five or more family members living in one household (4).

In a population-based survey in Bangladesh, the prevalence of lifetime sexual violence was 37 % and 50% in urban and rural areas, respectively, (5). Another study from Bangladesh reported that 49.4% of women experienced lifetime physical violence by their partners and 18.4% reported being sexually abused (1). Women who experienced controlling behavior by their husbands were more likely to experience physical abuse by their husbands (6).

A study from Iraq showed that violence against women was culture dependent. The rate of physical and/or sexual violence against women by intimate partners was 20.8% in the Christian culture while the rate was 18.8% in the Muslim culture (7). The rate of psychological violence was 40% in the Muslim culture, which was significantly higher than the prevalence (24.8%) of the Christian culture. The factors related to violence were alcoholic husbands and having children (7).

Domestic violence is the cause of 3.5% of the patients with maxillofacial fractures referred to a hospital in Tehran, Iran. One third of the husbands of the women with domestic violence were drug addicts (8).

Another study on mothers of children aged 6 to 18 months from Iran showed that the overall prevalence of emotional abuse, sexual abuse and physical abuse was 53.5%, 34.7% and 26.7%, respectively. Opium use by husband was a risk factor (9).

In another study from Iran, the rate of physical, psychological and sexual abuse against women was 43.7%, 82.6% and 30.9%, respectively (10). A study from Iran reported that women were abused physically (73.5%), emotionally (92.2%) and sexually (49.6%) for at least one time in their life (11). Regardless of gender, physical or psychological violence increases the risk of sexual violence (12).

Current explanatory study aims to survey the rate and predictors of abuse of wives by their inpatients psychiatric husbands. To the best of our knowledge, no published study has ever investigated this rate in the sample of inpatients husbands who suffer from psychiatric disorders.

## Materials and Method

***Method***

Participants of this survey were a convenient sample of wives of 209 male psychiatric inpatients in Shiraz, Iran. A self-reported questionnaire was used to gather the data; this valid questionnaire has been used in a previous study and its reliability was 0.8 (13). This questionnaire surveys domestic violence towards wives at home. It assesses physical, emotional, social and economic violence. In the current study, four questions about sexual abuse were added. 

The questions related to each type of violence are depicted in Table 1. For example, the questions about economic abuse were: “Not giving money to spend on house things”, and “Constant control over your spending”. Some of the questions about physical violence were: “Beating you or throwing things at you”, “Throwing things at you”, and “Slapping you”.

In total, there were 28 questions with Likert type responses. The response could range from “never’, “rarely”, “sometimes”, “usually” to always”. The number of questions asking physical, emotion, social and economic violence was 7, 5, 8, and 4, respectively. The total score ranged from 0 to 126. 

This study was approved by the Ethics Committee of Shiraz University of Medical Sciences. Those patients or wives who would not like to provide written informed consent were not included. 

Statistical Analysis

Several separate linear regression analyses, stepwise method, were performed to examine the association of the total score and subscale scores of abuse with the independent variables. The variables of wife’s age, age at marriage, duration of marriage, number of children, involuntary marriage, living in an extended family, education of wife, employment of wife, level of income of wife, substance use by wife, self-reported psychiatric problems in wife, self-reported physical problem in wife, husband’s age, age of husband, educational level of husband, employment of husband, substance use by husband and self-reported physical problem in husband were considered as independent variables. Education was categorized into those with diploma or less and those with higher than diploma. Occupation was categorized into two categories of being employed or unemployed. Monthly income was categorized into two income levels of less than 5,000,000 Rials or more than 5,000,000 Rials.

Pearson Correlation Coefficient was used to examine the correlation of different types of abuse scores. P value less than 0.05 was considered as statistically significant.

## Results

The educational level of wives was as follows: 24.3% had high school diploma, 20.4% had a bachelor’s degree, 17.5% had a master’s degree, 1.9% were illiterate, 13.6% had elementary school and 21.4% had secondary school; 78.2% of the wives had less than 5,000,000 Rials (Currency of Iran) self-reported monthly income. The basic characteristics of the participants are demonstrated in Table 2. The rate of addiction to opium and alcohol in wives was 1.5% and 8.7%, respectively. Of the patients, 38.3% had not used any substance. Table 2 illustrates the frequency of different types of abuse by the husbands. There were significant correlations between the different types of abuse (Table 2).

The rate of individuals who reported that their husbands have never socially abused them was very low. The range of items was from 2.5% to 17.5%. In other words, more than 80% of the husbands have socially abused their wives. For example, 53.7% of the subjects were frequently abused socially by their husbands (by humiliating them in front of others); 71% of the wives reported that they were regularly being threatened by their husbands; 43.9% of the wives were frequently kicked out of their home by their husbands.

Linear regression analysis revealed that none of the independent variables were associated with the score of social abuse.

As Table 2 demonstrates, all the forms of physical abuse are common. The percent of wives who selected the answer of “never” or “rarely” was 2.9% and14.6%, respectively. It means that the rest of wives selected the answers which ranged from “sometimes” to “always”. Of the subjects, 73.0% were regularly beaten by their husbands; 72.8% reported that their husbands break the house appliances or furniture and/or throw them towards their wives; 58.7% of the wives were slumped by their husbands sometimes or often always; 50.5% of the wives were frequently abused through pulling their hair.

Regression analysis showed that none of the independent variables predicted physical abuse score of wives by their husbands. 

Only 14.1% of the wives reported that their sexual relationship with their husbands is with desire; 26.2% of the wives reported that their sexual relationship with their husbands is forced by their husband; 59.7% of the sample reported lack of desire for sexual relationship with their husbands; 67.5% of the wives reported that their husbands tease and humiliate them during the sexual relationship. 

The score of sexual abuse was not statistically associated with either of the other variables.

The prevalence of the items related to economic abuse is displayed in Table 2. Of the subjects, 62.1% reported that their expenses were not frequently covered by their husbands, and only 10.2% of the wives were completely financially supported by their husbands. In addition, 66.0% of the husbands strictly controlled their wives’ expenses; 49.6% of the husbands did not regularly let their wives to know about their income. 

Regression analysis showed that wives’ income was only associated with the economic abuse score (odds ratio = -.17, P<0.03, 95% CI: -1.2 to -.06).

The score of economic abuse was not statistically associated with either of the other variables.

Of the sample, 77.2% reported that their husbands frequently humiliated and teased them (Table 1); 62% of the husbands frequently shouted or yelled; 31.1% of the wives were sometimes threatened by their husbands by a knife or gun; 45.8% of the wives were humiliated by their husbands because of their physical appearance from time to time. 

Regression results revealed that none of the independent variables was statistically associated with the psychological abuse score. None of the independent variables was statistically associated with total score of abuse. Table 3 displays that there are significant correlations between different types of domestic violence.

## Discussion

One of the most striking finding of this study was a very high rate of conducting different types of domestic violence by the psychiatric inpatients. These high rates are observed for the different types of abuse. These results suggest that wives of psychiatric inpatients are frequently victimized by their husbands socially, emotionally, physically, psychologically and even sexually. These results are in accordance with the results of studies conducted in community samples from Iran (11, 13) and other countries. However, no published study about the rate of abuse by psychiatric inpatient husband towards their wives was found to compare our results with theirs. It may suggest that violence against wives of psychiatric inpatients is a part of husbands’ psychiatric disorders. 

Another finding of this survey was lack of association of substance use and the score of abuse by husbands. This finding is not similar to reports from community samples (11, 14). A very high rate of violence may explain this finding. Moreover, there was a significant correlation between different types of abuse, suggesting that many types of abuse usually co-occur. There are some limitations needed to be considered. The sample was from a clinical sample from Shiraz. Therefore, generalization of results to other populations or settings should be made with caution. The only source of information was wives. No collateral information or source was included. 

Clinical Implication

The current results suggest a very high alarming rate of all types of abuse against women among a sample of wives of psychiatric inpatients in Shiraz, Iran. This high rate of violence and its association with substance abuse suggest the need for urgent planning to address them.

## Limitations

All the patients were from one university affiliated hospital. More over the results were based on the wives reported prevalence without any collateral informants. 

## Conclusion

It seems that the rate of domestic violence committed by husbands towards wives of patients with psychiatric problems is considerably high. This matter needs further attention in psychiatric management of these patients and their family members.

**Table 1:** Prevalence of Different Types of Abuses committed by the husbands

		**Never**	** Rarely**	**Sometimes**	**Usually**	**Always**
Economic	Not giving money for the expenses to the wife	21(10.2)	57(27.7)	107(51.9)	16(7.8)	5(2.4)
	Constant control over her expenses	6(2.9)	64(31.1)	113(54.9)	18(8.7)	5(2.4)
	Not telling her about his income	32(15.5)	72(35.0)	63(30.6)	30(14.6)	9(4.4)
	Opposing to her having a job	18(8.7)	61(29.6)	58(28.2)	47(22.8)	22(10.7)
Psychological	Rage, frowning and sour action	3(1.5)	44(21.4)	103(50.0)	49(23.8)	7(3.4)
	Shouting and yelling	3(1.5)	52(25.2)	96(46.6)	52(25.2)	3(1.5)
	Actions or comments against the wife	9(4.4)	52(25.2)	94(45.6)	41(19.9)	10(5.1)
	Humiliating her because of her physical appearance	29(14.1)	62(30.1)	74(35.9)	33(16.0)	8(3.9)
	Threatening her with a knife or a gun	41(19.9)	103(50.0)	28(13.6)	31(15.0)	3(1.5)
Social	Swearing and insulating	5(2.5)	36(17.7)	122(60.1)	32(15.8)	8(3.9)
	Making fun of her in front of others	17(8.4)	77(37.9)	81(39.9)	25(12.3)	3(1.5)
	Throwing her out of the house	35(17.2)	79(38.9)	64(31.5)	20(9.9)	5(2.5)
	Threatening and harming the people she likes	16(7.9)	71(35.0)	86(42.4)	29(14.3)	1(0.5)
	Preventing her from meeting her friends	5(2.5)	54(26.7)	105(52.0)	26(12.9)	12(5.9)
	Prohibiting her from meeting her parents and relatives	22(10.9)	78(38.6)	74(36.6)	25(12.4)	3(1.5)
	Locking her up in the house	10(4.9)	49(24.1)	96(47.3)	44(21.7)	4(2.0)
	Following her and spying on her	8(3.9)	73(36.0)	85(41.9)	32(15.5)	5(2.5)
Physical	Beating or throwing things at her	9(4.4)	46(22.3)	119(57.8)	22(10.7)	10(4.9)
	Breaking the household objects or throwing them at her	10(4.9)	46(22.3)	116(56.3)	25(12.1)	9(4.4)
	Throwing things at her	9(4.4)	46(22.4)	113(55.1)	26(12.7)	10(4.9)
	Slapping her	16(7.8)	69(33.5)	86(41.7)	29(14.1)	6(2.9)
	Pulling her hair	30(14.6)	72(35.0)	68(33.0)	30(14.6)	6(2.9)
	Hitting her with some objects	6(2.9)	73(35.4)	91(44.2)	28(13.6)	8(3.9)
	Beating her up with a belt or a stick	10(4.9)	73(35.4)	82(39.8)	31(15.0)	10(4.9)
Sexual	How is the wife’s sexual relationship with her husband	With desire	Without desire	Forced spouses		
		29(14.1)	123(59.7)	54(26.2)		
	Does he humiliate her during the sexual relationship?	23(11.2)	44(21.4)	107(51.9)	29(14.1)	3(1.5)
	Sex during menses	48(23.3)	75(36.4)	67(32.5)	13(6.3)	3(1.5)
	Sex when ill	71(34.5)	87(42.2)	37(18.0)	8(3.9)	3(1.5)

**Table 2:** The Basic Characteristics of the Participants

Age range of the husbands	21 to 62 years
Mean age of the husbands	35.4(7.1) years
Age range of the wives	19 to 54.0 years
Mean (SD) of the wives	31.0(5.9) years
Mean duration of their marriage	9.3(6.1) years
Mean (SD) age of the wives at marriage	21.4(3.7) years
Those without any child	14.5%
Those with one child	31.1%
Those with two children	29.1%
Those who were from the capital city of Shiraz	59.7%
Self-reported involuntary marriage	38.3%
Living in husbands’ extended family	30.1%
The rate of employment for wives	18.9%
Self- reported psychiatric problems in wives	12.1%

**Table 3:** Correlation between the Different Types of Abuse Scores indicating co-occurrences

		**Economy Score**	**Psychology ** **Score**	**Social Score**	**Physical Score**
sychology Score	Pearson Correlation	.138*			
	Sig. (2-tailed)	.048			
Social score	Pearson Correlation	.159*	.612**		
	Sig. (2-tailed)	.024	.000		
Physical score	Pearson Correlation	.126	.609**	.642**	
	Sig. (2-tailed)	.072	.000	.000	
Sexual score	Pearson Correlation	.084	.471**	.497**	.573**
	Sig. (2-tailed)	.230	.000	.000	.000

* P<0.05,

** P<0.001
